# Prevalence and Associated Risk Factors of Intestinal Parasites among Diarrheic Under-Five Children Attending Bahir Dar and Han Health Centers, Northwest Ethiopia: A Cross-Sectional Study

**DOI:** 10.1155/2022/7066529

**Published:** 2022-05-04

**Authors:** Jemal Mohammed, Alemtsehay Shiferaw, Abaynesh Zeleke, Yemsrach Eshetu, Zenash Gebeyehu, Animen Ayehu, Yesuf Adem

**Affiliations:** ^1^Amhara Regional Health Bureau, Bahir Dar, Ethiopia; ^2^Department of Medical Laboratory Sciences, School of Health Sciences, College of Medicine and Health Sciences, Bahir Dar University, Bahir Dar, Ethiopia

## Abstract

**Background:**

Intestinal parasitic infection is one of the major public health problems in developing countries including Ethiopia. The problem is highly pronounced in children under five years of age who are not able to adhere to prevention and control precautions.

**Objective:**

To assess the prevalence of intestinal parasitic infections (IPIs) and associated factors among diarrheic children under five years of age attending Bahir Dar and Han Health Centers, Bahir Dar, Northwest Ethiopia.

**Methods:**

An institution-based cross-sectional study was conducted among diarrheic children less than five years of age at Bahir Dar and Han Health Centers, Northwest Ethiopia, 2020. A total of 221 diarrheic children less than five years of age were recruited using the convenience sampling technique. Data were collected using a pretested structured Amharic version questionnaire after obtaining informed consent from children's parents or guardians. Approximately 1 mL of fresh fecal specimen was collected and subjected to direct saline wet mount microscopy. All data were entered into Epi Info version 7 and transferred to SPSS statistical software version 20 for analysis. Logistic regression was employed to assess risk factors associated with increased prevalence of intestinal parasitic infection in diarrheic children under five years of age, and a *P* value < 0.05 was taken as statistically significant.

**Results:**

The overall prevalence of intestinal parasites was 19% (95%CI = 14–24.6). The most prevalent intestinal parasites were *Entamoeba histolytica/Entamoeba dispar* (24 (10.86%)), *Giardia lamblia* (12 (5.43%)), and *Ascaris lumbricoides* (2 (0.9%)). Children's mother/guardian washed their hands sometimes after the toilet (AOR = 2.98, 95% CI: 1.09-8.18), children who always eat unwashed fruits and vegetables (AOR = 4.63, 95% CI: 1.09–19.75), and children's mother/guardian who had no knowledge about the mode of transmission (AOR = 4.03, 95% CI: 1.04–15.64) were risk factors significantly associated with increased intestinal parasitic infections.

**Conclusion:**

The prevalence of intestinal parasitic infections was found low compared to the population prevalence reported by WHO. However, strengthening health education about food and personal hygiene of both children and their mothers/guardians is crucial to limit the transmission of IPIs.

## 1. Background

A parasite is a living organism which lives in or upon another organism (host) and derives nutrients directly from it [1]. Intestinal parasitic infections (IPIs) are major public health problem worldwide, being most prevalent in developing countries where environmental and social factors contribute to the development of the diseases [2]. Parasitic intestinal protozoa together with soil-transmitted helminths are causes of morbidity, abdominal discomfort, mechanical irritation of intestinal mucosa, malabsorption syndromes, and often mortality in tropical and subtropical regions around the world [3].

Globally, intestinal parasites are distributed worldwide, and it is estimated that 3.5 billion people are affected by IPIs and 450 million are ill because of these infections. Moreover, the prevalence of IPIs is the highest in poor and developing countries [4]. Soil-transmitted helminths (STHs) including *Ascaris lumbricoides* (*A. lumbricoides*), *Trichuris trichiura* (*T. trichiura*), and hookworms are estimated to affect more than 25% of the world's population [5]. Protozoan parasites which commonly cause disease in the intestine are *Giardia lamblia* (*G. lamblia*) and *Entamoeba histolytica* (*E. histolytica*). *Giardia lamblia* is the most prevalent intestinal protozoan parasite with high worldwide distribution. Ten to 50% prevalence rates of giardiasis in children were reported from tropical regions, and greater than 250 million people are infected worldwide. The global burden of amoebiasis is approximately 400,000 cases annually and accounting for 40,000–110,000 deaths next to malaria and schistosomiasis [6].

Intestinal parasitic infections are very common in Ethiopia, and the magnitude of infection varies from place to place. Intestinal parasitic infections account for the second most predominant causes of outpatient morbidity in the country [7]. High prevalence of parasitic infections in Ethiopia could be due to unsafe and inadequate provision of water, unhygienic living conditions, and the absence of proper utilization of latrine [8]. Although people of all ages can be infected with intestinal protozoa and helminths, preschool children are the most vulnerable group because of the immaturity of their immune system, inherent rudimentary hygiene habits, frequent contact with contaminated environments, and high frequency of interpersonal child to child contact [9]. In this group of individuals, enteroparasites are known to be responsible for high morbidity with diarrhea, malnutrition, and several physiologic abnormalities [10]. Playing with soil, sucking fingers, and defecation in open field are among the most frequently indicated reasons for the highest prevalence of IPIs in preschool children. Maternal awareness for the prevention and control of intestinal parasite has also its own impact on the prevalence of IPIs. Access to safe water, sanitation, and health education is central to reduce the impact of intestinal parasites [11].

More than a billion people suffer from both protozoan and helminth parasitic infections. In Ethiopia, the overall national prevalence of any helminth infection was 29.8% with variable degree of prevalence among regions. Intestinal parasites cause morbidity and mortality outcome of human disease [12]. The severity of IPIs worsens among patients with chronic illnesses, elderly patients, and preschool children due to their susceptibility to high parasitic load. In preschool children, intestinal parasitic infection is also one of the causes of impaired childhood growth and cognitive development and also contributes to deaths [13].

More than 266 million preschool-aged and 609 million school-aged children in 106 countries are estimated in need of preventive chemotherapy for soil-transmitted helminths. In Africa, more than 13.8 million preschool-aged children in need of treatment were treated. In Ethiopia, the main strategies of IPI prevention and control are mass drug administration, case detection, and transmission control [14]. In most Ethiopian cities including Bahir Dar, several factors contribute to the prevalence of intestinal parasite including shortage of clean water supply, open field defecation, and poor sanitation practices. Frequent use of untreated waste water streams for urban agricultural irrigation, uncooked vegetables [15], children's untrimmed fingernails, mothers' poor hand hygiene, and drinking water from rivers [16] may transmit IPIs and increase the prevalence of intestinal parasites. Furthermore, lack of data regarding the prevalence of intestinal parasites in diarrheic children and associated risk factors in the study area overlay the problem. Therefore, this study was aimed at assessing the prevalence of intestinal parasite infections and associated risk factors among children less than five years of age attending in Bahir Dar and Han Health Centers, Northwest Ethiopia.

## 2. Materials and Methods

### 2.1. Study Area

The study was conducted in children less than five years of age at Bahir Dar and Han Health Centers, Bahir Dar City. Bahir Dar is one of the fast-growing cities in the country and serves as the capital city of Amhara National Regional State which is located 563 km away from the northwest of Addis Ababa, the capital city of Ethiopia. The northern part of the city is surrounded by Lake Tana, which is the largest lake in Ethiopia and the source of the Blue Nile River. The Blue Nile River crosses the city and then surrounds the eastern part of the city. According to the 2007 Ethiopian population census, the total population of the city is about 180,094 with the majority of them are children. The city had seven hospitals, ten health centers, and different private health institutions (Bahir Dar City Administration Office).

### 2.2. Study Design and Period

An institution based cross-sectional study design was carried out from January 18, 2020 to February 30, 2020.

### 2.3. Population

#### 2.3.1. Source Population

The source population of this study was all children under five years of age who visited Bahir Dar and Han Health Centers during the study period.

#### 2.3.2. Study Population

The study population was all diarrheic children under five years of age who are suspected of intestinal parasitic infections.

#### 2.3.3. Study Subjects

All conveniently selected diarrheic children under five years of age were study subjects.

### 2.4. Inclusion and Exclusion Criteria

#### 2.4.1. Inclusion Criteria

Children less than five years old who had diarrhea were included in the study.

#### 2.4.2. Exclusion Criteria

Children who took standard intestinal parasite treatment and children who had serious diseases for the last two weeks were excluded from the study.

### 2.5. Study Variables

#### 2.5.1. Dependent Variable

- Prevalence of intestinal parasite

#### 2.5.2. Independent Variables

- Sociodemographic characteristics

- Age, sex, family residence, family occupation, mother/guardian educational status, family income

(ii) Behavioral and environmental risk factors

- Dining utensil cleanness, hand washing after toilet before touching child, source of drinking water, finger nail status of child, type of toilet, child playing ground, knowledge of mother on IP transmission, family history of IP infection, child took other food before the age of six months, child meal, child eats unwashed fruits and vegetables

### 2.6. Sample Size Determination

The sample size was determined by using single population proportion formula. A prevalence of 15.5% was taken from study done in Dessie Referral Hospital (15.5%) [17]. Accordingly, the following formula is used to calculate the sample size:

(1) $$\begin{array}{c} n=\frac{\left(Z^{2}\alpha /{}_{2}P\left(1-P\right)\right)\;}{d^{2}}, \end{array}$$

where *n* is the number of study subjects enrolled in the study, *Z* is the 95% confidence interval = 1.96, *d* is the marginal error between the samples and the population = 0.05, and *P* is the prevalence of IP = 15.5%.

Therefore,

(2) $$\begin{array}{c} n=\frac{{\left(1.96\right)}^{2*}{\left(0.155\right)}^{*}\left(0.845\right)}{{\left(0.05\right)}^{2}},\\ n=201. \end{array}$$

Considering a nonresponse rate of 10%, the final sample size was 221.

### 2.7. Sampling Technique

A convenient sampling technique was used to enroll study participants attending at Bahir Dar and Han Health Centers during the study period who fulfilled the inclusion criteria.

### 2.8. Data Collection and Processing

Data were collected using pretested structured Amharic version questionnaire. Data collection was carried out by principal investigators and coinvestigators. At each data collection spot, sufficient explanation about the aim of the research was given to the parents/guardians before the interview was carried out. A single, approximately 1 mL of fresh stool was collected from study participants in clean, labeled stool cups. Direct saline wet mount of stool specimen was prepared and examined under microscopy by two experienced laboratory technicians.

### 2.9. Quality Control

To ensure quality control, the questioner was pretested on 5% of the actual sample size at Meshenti health center. The laboratory procedures including collection, handling, and examination of specimens were performed according to standard operating procedures. Standardized stool cup was used to ensure good sample quality and convenient sample collection. To ensure the validity of 0.85% sodium chloride (NaCl), microscopic examination was checked for the presence of impurities. Finally, to ensure the quality of the investigation, two readers examined the slides independently and their reading was compared. Discordant was immediately resolved with a discussion of each other and in consultation with other experts.

### 2.10. Data Management and Analysis

All data were registered in research data sheet during the study period which were entered into epidemiological information statistical software (Epi Info) version 7 and transferred to Statistical Package for Social Sciences (SPSS) statistical software version 20 for analysis. Logistic regression was used to evaluate risk factors. Then, study findings were explained in words, tables, and other statistical summary techniques. *P* value less than 0.05 was taken as statistically significant.

### 2.11. Ethical Consideration

Ethical clearance was obtained from the Research and Ethics Committee of College of Medicine and Health Sciences, Bahir Dar University (CMHS/625/2020), and permissions were obtained from Bahir Dar and Han Health Centers. After brief explanation of the study purpose, benefits, and possible risks of the study, informed assent was obtained from the study participant's family/guardian. Specimen collection, processing, and examination procedures adhered to the standard protocols prepared by the department of Medical Laboratory Sciences, CMHS-BDU. Confidentiality and any special data security requirements were maintained and assured by using code numbers. Results of the laboratory examinations that have a direct benefit in the health of the study participants were informed to assigned clinicians and the participants get their results and treatment duly as required.

## 3. Results

### 3.1. Sociodemographic Characteristics of Study Participants

A total of 221 under-five children (116 males and 105 females) were enrolled in this study. Of the total, 42.7% of the children were under the age group of 24-59 months. Majority of the children's mothers were merchants. Around 28% of children's mother had diploma and above educational status, and children who had mothers that are unable to read and write were more affected by intestinal parasite (12/42) (Table 1).

### 3.2. Prevalence of Intestinal Parasite among Diarrheic Children under Five Years of Age

In this institution-based cross-sectional study, a total of 221 diarrheic children under five years of age were recruited; 42 (19%) (CI: 14%-24%) were positive for at least one parasite. *E. histolytica/E. dispar* (24 (10.86%)), *G. lamblia* (12 (5.43%)), and *A. lumbricoides* (2 (0.9%)) were identified among the study participants. Intestinal helminthic infection accounted the least (2/42), while intestinal protozoal parasitic infection was high and accounted for 95.2% (40/42). Among the identified intestinal parasite species, the most frequent parasite was *E. histolytica/E. dispar* (24 (10.86%)) followed by *G. lamblia* (12 (5.43%)) and *A. lumbricoides* (2 (0.9%)).

**Figure 1:**
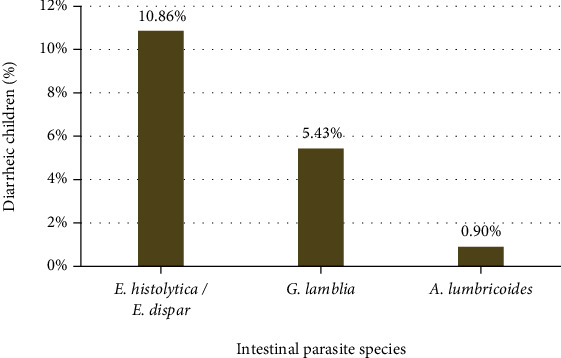


**Figure 2:**
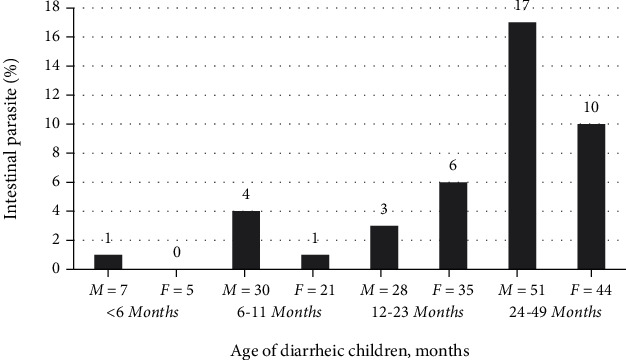


While single infections are common, double coinfection (*E. histolytica* and *G. lamblia*) was also observed in 4 (1.8%) children. However, triple infection was not detected among the study participants (Figure 1). In this cross-sectional study, male children under five years of age were more affected than female children by intestinal parasites. The most affected age group was 24 to 59 months (Figure 2).

### 3.3. Risk Factors Associated with Intestinal Parasitic Infection

In this cross-sectional study, different possible risk factors were assessed using bivariable and multivariable logistic regression analysis (Table 2). Risk factors such as eating unwashed fruits/vegetable (AOR = 4.63, 95% CI: 1.09-19-75, *P* = 0.038), mother/guardian infrequent hand washing after toilet (AOR = 2.98, 95% CI: 1.09-8.18, *P* = 0.034), and mother/guardian with no knowledge about IP mode of transmission (AOR = 4.03, 95% CI: 1.04-15.64, *P* = 0.044) were found to be significantly associated with the prevalence of intestinal parasitic infections in children. Based on the above facts, children who frequently eat unwashed vegetable/fruits were more likely to be infected with intestinal parasitic infection as compared to the children who eat washed vegetables. Similarly, infrequent washing of mothers' hands after toilet was more likely to expose their children to intestinal parasitic infection as compared to mothers with frequent washing of hand after toilet. Mothers/guardians with no knowledge about mode of transmission of IPIs were more likely to infect their children with intestinal parasites as compared to mothers who had knowledge about mode of transmission of IPIs.

## 4. Discussion

It is of paramount importance to know the distribution and extent of intestinal parasitic infection in a given community to devise successful preventive and therapeutic interventions. This study assessed the prevalence and risk factors associated with intestinal parasite infections. The overall prevalence of intestinal parasitic infections among diarrheic children under five years of age was 19% (95% CI: 14–24%). This finding is comparable with previous studies done at Woreta Health Center (18.7%) [18], Debre Birhan Referral Hospital (17.4%) [19], and Dessie Referral Hospital (15.5%) [17]. Intestinal parasite infections are more prevalent among the poor sections of the population and closely linked with low economic level, poor personal and environmental sanitation, overcrowding, limited access to clean water, tropical climate, and low altitude [20].

However, the current study showed a lower prevalence of intestinal parasite infection as compared to those studies conducted at Adare hospital in Hawassa town (26.6%) [21] and Northern Mozambique (31.6%) [22]. This could be due to the fact that only saline wet mount diagnostic method was used in the current study while studies in Port Blair hospital, Northern Mozambique, and Adare hospital in Hawassa town, Ethiopia employed combinations of diagnostic methods such as saline wet mount and Lugol's iodine, concentration technique, and acid fast staining. On the contrary, the current prevalence of intestinal parasite infection among children under five years of age is higher than the prevalence reported from Debre Birhan town, North Shoa, Ethiopia (9.8%) [23], and Gondar University, Gondar, Ethiopia (9.02%) [24]. This difference might be due to geographical location, climate difference, and time of survey.

*E. histolytica*, with a prevalence rate of 10.86%, was the most frequently encountered parasite in this study. This finding is supported by studies conducted in Hawassa (11.4%) [21] and Dessie Referral Hospitals (6.5%), Ethiopia [17] while it was lower than the prevalence rates reported from Woreta health center (18%), Ethiopia [18] and Port Blair hospital (21%), India [25]. The difference might be due to various reasons which include methods of diagnosis, difference in geographical settings, sanitation facility coverage, accessibility of safe water, and personal hygiene dissimilarities. In this study, *G. lamblia* with a prevalence rate of 5.43% was the second predominant intestinal parasite while it was the predominant intestinal parasite reported by a study conducted in Debre Birhan, Ethiopia [19]. On the other hand, the highest prevalence rate of *A. lumbricoides* was reported by studies conducted at Port Blair hospital (30.5%), India [25] and North Mozambique (13.1%) [22]. The species distribution difference might be due to diagnostic tools used, socioeconomic status of children's parents/guardians, and geographical area difference. Double infection (*E. histolytica* and *G. lamblia* 4 (1.8%)) was also recorded. This finding was comparable with the study conducted at Dessie Referral Hospital, Ethiopia [17]. Enteric infections in young children are often caused by several aetiologic agents including protists, bacteria, and viruses in either single or coinfections. Single enteric infections with bacterial species were not common in children aged below five years [26] whereas higher prevalence of coinfections with two enteric pathogens in diarrheic children was indicated previously [26, 27]. Contrary to our results, the common coinfection of protists were caused by *E. histolytica* and *Blastocystis* species wherever single infections with *Blastocystis* species were higher than single infections with *E. histolytica* [28].

In the present study, different risk factors contributing to increased prevalence of IPIs in the study area were analyzed using multivariate analysis. Frequent eating of unwashed vegetables and fruits was significantly associated with increased prevalence of IPIs. Children less than five years of age who ate unwashed fruits and vegetables were 4.63 times at higher risk to be infected by IPs than those who did not eat unwashed fruits and vegetables. This might be due to the contamination of fruits and vegetable with the infective stages of IPs and children under five years of age easily ingest them. In addition, intestinal parasitic infection accounts for a global health burden in developing countries mainly due to fecal contamination of water and food and climatic, environmental, and sociocultural factors [29–31].

Irregular hand washing practice of mothers/guardians after toilet before touching the child was also found to be risks for increased prevalence of intestinal parasites among children. Children whose mother/guardian washed their hands occasionally after toilet before touching the child were at higher risk to be infected by IPs than children whose mothers/guardians washed their hands frequently after toilet before touching the child. This study finding is supported by a study conducted in Hawassa, Ethiopia [21]. This is because of the fact that parasites are commonly in contaminated toilets and other unhygienic areas. So, contamination of hands during defecation and poor hand washing practice increased the transmission of parasites from mothers/guardians to the child. The study also showed that no knowledge of mother/guardian about mode of transmission of intestinal parasite was a statistically significant risk factor for the increased prevalence of IPIs. In addition, children whose mother/guardian had no knowledge on mode of transmission were 4 times at higher risk to be infected by IPs than whose mother/guardian had knowledge on mode of transmission of IPs.

## 5. Limitations of the Study

The limitation of this study was unable to perform other diagnostic methods such as concentration techniques, modified acid fast staining, and Lugol's iodine. Repeated stool examinations for some parasites with intermittent occurrence of diagnostic stages were not carried out.

## 6. Conclusion

Intestinal parasitic infection among children less than five years of age was found to be low compared to the population prevalence of WHO annual report. Most study participants were affected by single IPs. *E. histolytica* was found to be the most predominant intestinal parasite species. Risk factors statistically associated with increased prevalence of IP infection were eating unwashed vegetables/fruits, mother's/guardian's hand washing after toilet before touching a child at some instants only, and mother/guardian with no knowledge about mode of transmission of intestinal parasites. Health education about food, environmental and personal hygiene, and transmission mode of intestinal parasites will contribute to decrease the prevalence of IPIs in the study area [21].

## Figures and Tables

**Table 1:** Sociodemographic characteristics of study participants (*n* = 221) at Bahir Dar and Han Health Centers, Northwest Ethiopia, 2020.

Variables	Characteristics	Frequency (%)	Positive for IPIs	
			Number	%
Sex	Male	116 (52.5)	25	11.3
	Female	105 (47.5)	17	7.7
Age	<6 months	12 (5.9)	1	0.45
	6-11 months	51 (23.5)	5	2.26
	12-23 months	63 (28.1)	9	4.07
	24-59 months	95 (42.5)	27	12.2
Family residence	Urban	155 (70.1)	24	57.1
	Rural	66 (29.8)	18	42.9
Mother/guardian educational level	Not able to read and write	38 (17.1)	12	28.6
	Able to read and write	58 (26.2)	11	26.2
	Grade 1-8	28 (12.6)	6	14.3
	Grade 9-12	34 (15.3)	5	11.9
	Diploma and above	63 (28.5)	8	19.0
Mother's/guardian's occupation	Farmer	56 (25.3)	16	38.1
	Merchant	71 (32.1)	12	28.6
	Employee	68 (30.7)	8	19.0
	Housewife	26 (11.7)	6	14.3
Family monthly income	<1000 birr	63 (28.5)	18	42.8
	1000-3000 birr	77 (34.8)	12	28.6
	>3000 birr	81 (36.6)	12	28.6

**Table 2:** Bivariable and multivariable logistic regression analysis of risk factors for IPIs among Bahir Dar and Han Health Centers, Northwest Ethiopia, 2020.

Variable	Characteristics	Intestinal parasitic infection		Statistics		
		Positive *N* (%)	Negative *N* (%)	Crude odds ratio (95% CI)	Adjusted odds ratio (95% CI)	*P* value
(a) Sociodemographic risk factors						
Sex	Male	25 (11.3)	91 (41.2)	1	—	—
	Female	17 (7.7)	88 (39.8)	0.70 (0.35-1.39)	—	—
Age	<6 months	1 (0.4)	11 (5.0)	1	1	—
	6-11 months	5 (2.3)	46 (20.8)	1.19 (0.13-11.29)	0.95 (0.08-11.75)	0.97
	12-23 months	9 (4.1)	54 (24.4)	1.83 (0.21-15.98)	1.21 (0.09-15.30)	0.88
	24-59 months	27 (12.2)	68 (30.8)	4.37 (0.54-35.49)^∗^	3.66 (0.31-42.99)	0.30
Residence	Urban	24 (10.9)	131 (59.3)	1	1	—
	Rural	18 (8.1)	48 (21.7)	2.05 (1.02-4.10)^∗^	0.56 (0.16-1.96)	0.36
Mother/guardian educational level	Not able to read and write	12 (5.4)	26 (11.8)	3.13 (1.06-8.70)^∗^	2.53 (0.43-14.85)	0.30
	Able to read and write	11 (5.0)	47 (21.3)	1.61 (0.59-4.33)	1.53 (0.44-5.32)	0.50
	Grade 1-8	6 (2.7)	22 (9.9)	1.87 (0.58-6.03)	1.46 (0.26-8.22)	0.60
	Grade 9-12	5 (2.3)	29 (13.1)	1.18 (0.35-3.95)	0.86 (0.18-4.07)	0.85
	Diploma and above	8 (3.6)	55 (24.9)	1	1	—
Mother's/guardian's occupation	Farmer	16 (7.2)	40 (18.1)	1.33 (0.45-3.93)	0.37 (0.08-1.72)	0.20
	Merchant	12 (5.5)	59 (26.7)	0.68 (0.22-2.04)	0.40 (0.08-2.01)	0.26
	Employee	8 (3.6)	60 (27.1)	0.44 (0.14-1.44)^∗^	0.39 (0.08-1.98)	0.25
	Housewife	6 (2.7)	20 (9.0)	1	1	—
Family monthly income	<1000 birr	18 (8.0)	45 (20.4)	2.30 (1.01-5.23)^∗^	0.86 (0.16-4.07)	0.85
	1000-3000 birr	12 (5.5)	65 (29.4)	1.06 (0.44-2.53)	1.25 (0.43-3.63)	0.67
	>3000 birr	12 (5.5)	69 (31.2)	1	1	—
(b) Behavioral and environmental risk factors						
Mother/guardian hand washing habit after toilet	Always	26 (11.8)	148 (67.0)	1	1	—
	Sometimes	16 (7.2)	31 (14.0)	2.94 (1.41-6.12)^∗^	2.98 (1.09-8.18)	0.034^∗∗^
Child eating habit of unwashed vegetable and fruits	Always	6 (2.7)	9 (4.1)	4.3 (1.32-13.89)^∗^	4.63 (1.09-19.75)	0.038^∗∗^
	Sometimes	22 (10.0)	80 (36.2)	1.77 (0.85-3.69)^∗^	1.15 (0.43-3.08)	0.78
	Never	14 (6.3)	90 (40.7)	1	1	—
Freshness of child meal	Always fresh	6 (2.7)	44 (19.9)	1	1	—
	Sometimes fresh	32 (14.5)	125 (56.6)	1.88 (0.73-4.79)^∗^	0.94 (0.29-3.04)	0.92
	Rarely fresh	4 (1.8)	10 (4.5)	2.93 (0.69-12.37)^∗^	1.04 (0.18-5.93)	0.96
Child nail trimming habit	Always	28 (12.7)	148 (67.0)	1	1	—
	Sometimes	14 (6.3)	31 (14.0)	2.39 (1.13-5.05)^∗^	1.57 (0.56-4.41)	0.39
Child's playing ground	Clean	24 (10.9)	67 (30.3)	1	1	—
	Not clean	18 (8.1)	112 (50.7)	2.23 (1.13-4.41)^∗^	1.02 (0.42-2.46)	0.96
Source of drinking water of the family	Pump	40 (18.1)	162 (73.3)	1	—	—
	Spring	2 (0.9)	17 (7.7)	0.48 (0.11-2.15)	—	—
Type of toilet used in the family	Open field	11 (5.0)	30 (13.6)	1.93 (0.86-4.33)^∗^	1.32 (0.52-3.37)	0.56
	Public latrine	5 (2.2)	12 (5.4)	2.19 (0.71-6.76)^∗^	2.90 (0.68-12.47)	0.15
	Private toilet	26 (11.80)	137 (62.0)	1	1	—
Mother's/guardian's knowledge about mode of transmission	Yes	35 (15.8)	170 (66.9)	1	1	
	No	7 (3.2)	9 (4.1)	3.78 (1.32-10/82)^∗^	4.03 (1.04-15.64)	0.044^∗∗^

*N* = number; CI = confidence interval; ∗ = risk factors candidate by bivariable logistic regression analysis for multivariable logistic regression; ∗∗ = risk factors statistically significant at <0.05 *P* value for the increased prevalence of IPIs among children under five years of age.

## Data Availability

The findings of this study were generated from the original data collected during the study period and analyzed based on the stated methods and materials. The original data used to support the findings of this study will be available at any time upon request.

## Abbreviations

IPIs: Intestinal parasitic infections
STH: Soil-transmitted helminth
WHO: World Health Organization
SPSS: Statistical Package for Social Sciences.
